# Incidence of pregnancy loss and characterization of fetal development in red pandas

**DOI:** 10.1530/RAF-21-0079

**Published:** 2021-10-21

**Authors:** Jenna Lowe, Erin Curry

**Affiliations:** 1 Center for Conservation and Research of Endangered Wildlife, Cincinnati Zoo & Botanical Garden, Cincinnati, Ohio, USA

**Keywords:** carnivore reproduction, delayed implantation, embryonic diapause, wildlife, pseudopregnancy, ultrasonography

## Abstract

Previous reports indicate that red pandas (*Ailurus fulgens styani*) may experience fetal loss during gestation; however, neither the rate nor timing of pregnancy failure has been described in this species. The objective of this study was to utilize ultrasound video and images collected between 2010 and 2020 at the Cincinnati Zoo and Botanical Garden to better characterize pregnancy loss and fetal development. Trans-abdominal ultrasound examinations were performed on six female red pandas over a 10-year period, resulting in 12 profiles. Pregnancy was diagnosed via ultrasound in 10 of 12 profiles, and 40.0% of pregnancies showed evidence of fetal loss prior to parturition. Pregnancy loss was classified into lost (2 of 10; 20.0%), in which no cubs were produced, or partial loss (2 of 10; 20.0%), in which two concepti were visualized via ultrasound, but only one cub was born. Fetal loss occurred between days 51 and 23 pre-partum. Fetal growth characteristics were documented, including skeletal ossification (occurring between days 32 and 27 pre-partum), crown-rump length, head length, cranial length, and fetal heart rate (173–206 b.p.m.). These findings provide novel insights into pregnancy loss, may serve as a reference for milestones of fetal development, and may be useful in diagnosing pregnancy and assessing pregnancy loss in red pandas.

**Lay summary:**

For many wildlife species, there is no non-invasive method of determining pregnancy; therefore, the rate of pregnancy loss oftentimes is unknown. Many red pandas in human care that are paired for breeding are observed exhibiting normal mating behaviors; however, only a relatively low proportion of females produce cubs. We utilized animals conditioned for ultrasound examination to diagnose pregnancy and characterize the incidence and timing of pregnancy loss. In total, 12 potential pregnancies were monitored, beginning after breeding season and ending ~2 weeks prior to anticipated cubbing. Of these, ten were (83.3%) were diagnosed as pregnant, with 40% undergoing either full or partial pregnancy loss. Fetal growth characteristics, such as body length and head size, are described which may be useful for monitoring pregnancies and estimating fetal age. Results of this study provide novel data on pregnancy loss in red pandas. Insights into the rate and timing of reproductive failure may illuminate causes and contributing factors, ultimately allowing for improvements in husbandry which may result in greater reproductive success of individuals recommended for breeding.

## Introduction

Wild populations of red pandas (*Ailurusfulgensfulgens* and *Ailurusfulgensstyani*) have declined by 50% in recent years, primarily due to habitat loss, hunting, and poaching (Choudhury 2001, Jha 2011, Wei *et al.* 2005). Consequently, in 2015, the International Union for Conservation of Nature (IUCN) upgraded the status of the species from vulnerable to endangered (Glatston *et al.* 2015). *Ex situ* populations of red pandas may serve as insurance against species extinction and additionally, play a vital role in raising awareness of threats faced by their *in situ* counterparts. In zoos, the *A. f. styani* subspecies consists of approximately 370 individuals in the global population and 90 individuals managed by the regional North American Species Survival Plan (SSP). To maximize genetic diversity and prevent inbreeding, each year the SSP formulates strategic breeding recommendations; however, less than half of the recommended pairings result in the production of cubs (Glass *et al.* 2020). Neither the reasons for reproductive failure nor the point of reproductive malfunction are known, which in part is due to a paucity of methods to diagnose and monitor pregnancy in this species.

In the Northern hemisphere, red pandas breed in the late winter, usually January through March, and are thought to be induced ovulators (Spanner *et al.* 1997). Total gestation length ranges from 98 to 162 days (d) (Roberts & Kessler 1979, MacDonald *et al.* 2005, Budithi *et al.* 2016) due to the variable period of embryonic diapause, during which the embryo undergoes a developmental arrest lasting ~40 to 85 d (Roberts & Kessler 1979, MacDonald *et al.* 2005). Termination of diapause and resumption of embryo growth are likely controlled by photoperiod, similar to mink (Murphy & James 1974). In *A. f. styani*, fecal hormone analysis indicated that a secondary increase in progestogens occurred ~70 d prior to parturition (Curry *et al.* 2017), probably reflecting reactivation of the corpus luteum (CL), which initiates a cascade of events, including the termination of diapause, resumption of embryo growth, implantation, placentation, and fetal growth. Unlike diapause, the duration of placental pregnancy is fixed and is presumed to last ~60 to 70 d in red pandas. To date, the preponderance of research aimed at understanding pregnancy in this species has relied on fecal hormone analyses, largely progestogens to assess pregnancy status; however, several studies concurred that progesterone concentrations and patterns are not different in females that produced cubs compared to those that did not (MacDonald *et al.* 2005, Wei *et al.* 2005, Budithi *et al.* 2016, Curry *et al.* 2017). Our lab previously demonstrated that ultrasonography is an effective method to diagnose and monitor pregnancy in *A. f. styani* and, in one pregnancy, enabled the documentation of fetal loss post-implantation (Curry *et al.* 2017); however, the overall rate of pregnancy loss has not been described.

The Cincinnati Zoo and Botanical Garden (CZBG; Cincinnati, OH) boasts a successful history of managing breeding pairs of red pandas (*A. f. styani*) and has produced 97 cubs between 1983 and 2020. Since 2010, scientists at the Center for Conservation and Research of Endangered Wildlife (CREW) at CZBG have been conducting ultrasound examinations on female pandas housed at CZBG that were recommended for breeding by the SSP. Utilizing archived ultrasound videos and images obtained during voluntary examinations, the objectives of this study were to: (1) ascertain the rate of fetal loss; (2) resolve the timing of loss; and (3) better characterize fetal development. Insights into the rate and timing of reproductive failure may illuminate causes and contributing factors, ultimately allowing for improvements in husbandry which may result in greater reproductive success of individuals recommended for breeding. Establishing baseline data on fetal growth may permit animal care staff to gauge fetal age and serve as a reference for monitoring development.

## Materials and methods

### Animal use statement

The CZBG’s Institutional Animal Care and Use Committee (IACUC) approved the activities described in this study under protocols #14-119, *Ultrasound examination of the reproductive structures of red panda*s and #18-149, *Use of ultrasonography to detect and monitor pregnancy in non-domestic species*.

### Animals

Female red pandas (*A. f. styani*; *n*  = 6) were housed at CZBG between 2010 and 2020 and were paired for breeding as recommended by the Red Panda SSP (Table 1). Females were monitored for 2.00 ± 0.37 (mean ± s.e.m.) years with a range of 1**–**3 years per female, resulting in 12 profiles (profiles 1–12). Breeding (if observed) and parturition dates were recorded by animal care staff. At the end of each birthing season, profiles were categorized as parturient (*n* = 8) or non-parturient (*n* = 4) based on the production of cubs. Pregnancy duration was calculated from the last observed breeding date, if available, to the parturition date.

**Table 1:** Details of red panda profiles.

Profile ID	Status	SB#	Maternal age (years)	Year	Breeding date (s)	Parturition date	No. of cubs via ultrasound/No. of cubs birthed	Pregnancy duration days (d)	Range (d) of ultrasound exams pre-partum (# scans)
1	Term	0806	4.0	2012	Feb 25	Jun 11	1/1	107	101**–**27 (15)
2	Term	0806	5.0	2013	Feb 12, Feb 24	Jun 16	2/2	112	107**–**26 (16)
3	Term	0806	7.0	2015	Feb 1, Feb 11	Jun 13	2/2	122	87**–**31 (12)
4	Term	1319	2.0	2015	Feb 6, Feb 18	Jun 19	1/1	121	91**–**42 (10)
5	Term	1607	3.0	2019	n.o.	Jun 9	2/2	unk	39, 26 (2)
6	Term	1319	6.1	2019	n.o.	Jul 5	2/2	unk	65**–**37 (3)
7	PL	0257	8.0	2010	Jan 7–8	Jun 6	2/1	149	90**–**9 (11)
8	PL	1319	7.0	2020	n.o.	Jun 23	2/1	unk	37**–**14 (3)
9	Lost	0302	7.0	2010	Feb 23	n/a	1/0	unk	30**–**19 (3)*
10	Lost	0257	9.0	2011	n.o.	n/a	1/0	unk	51, 37 (2)*
11	Pseudo	0605	6.0	2012	Mar 22	n/a	0/0	n/a	120**–**11 (17)*
12	Pseudo	0605	7.0	2013	n.o.	n/a	0/0	n/a	107**–**26 (15)*

*In profiles classified as lost or pseudopregnant, day pre-partum was calculated based on mean parturition date of cohort.

n/a, not applicable; n.o., not observed; PL, partial loss; SB#, international studbook number; unk, unknown.

### Ultrasonography

Individuals were trained to stand voluntarily for percutaneous ultrasonography through desensitization to ultrasound gel/abdominal contact and operant conditioning with positive reinforcement using preferred food items. For examinations occurring between 2010 and 2015, a Sonosite Titan with a 4–2 MHz curved array transducer (Sonosite, Inc., Bothwell, WA) was employed, and in 2019–2020, an Ibex Pro with an MC8E transducer (6–10 MHz; EI Medical Imaging, Loveland, CO) was used. Although ultrasound exams were performed in 2016–2018, they were not recorded; therefore, they are not included in this dataset. Animals either stood on four feet or balanced bipedally against a wooden stand; anecdotally, animal position did not seem to impact examination success. During each examination, ultrasound gel was applied liberally to the abdomen or flank. The transducer was placed midline, approximately 6–8 cm cranial to the vulva and then swept laterally and cranially until a uterine horn was identified, starting on either the right or left and then repeating on the contralateral side. Regular examinations tended to commence in April, but the number, frequency, and timing of examinations varied by year (Table 1) and usually ceased approximately 2–4 weeks prior to anticipated parturition to reduce disturbances during nesting. Examinations were performed by two sonographers, and video and still images were saved from each examination.

**Figure 1:**
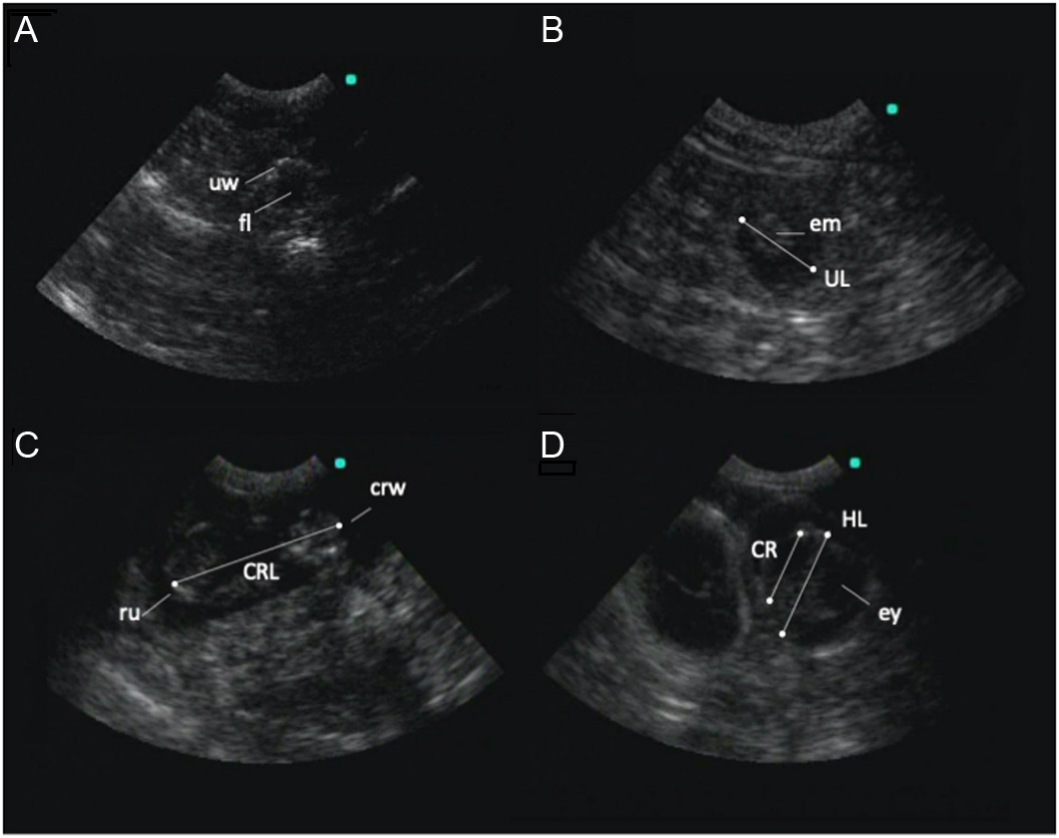
Trans-abdominal ultrasound images at d 75 (A), d 40 (B), d 27 (C), and d 23 (D) pre-partum. Panel A shows uterine wall (uw) and anechoic uterine fluid (fl). Panel B shows an embryo (em) and the uterine diameter measurement (UL). Panel C shows a developing fetus with crown (crw), rump (ru), and crown-rump length measurement (CRL). The image in panel D shows a twin pregnancy with two separate gestational sacs; the right sac shows a view of a fetus’ head and visible eye cavity (ey) with the head length (HL) and cranial length (CR) measurements.

Pregnancy was characterized by the presence of a fluid-filled gestational sac containing defined echogenic tissue, presumably of embryonic origin. The uterine lumen was defined as the anechoic cavity within the uterine horn and was measured as the diameter (cm) from the inner wall of one side of the horn to the inner wall of the opposing side (Fig. 1). When possible, fetal measurements were obtained, including head length (occipital bone to the tip of nasal bone), cranial length (occipital bone to frontal bone), and crown-rump length (length of cranial to the caudal margin of fetus) (Fig. 1). Ossification was defined as the transition of the skeleton to hyperechoic tissue that appeared denser than the surrounding soft tissue. Fetal heart rate was obtained when the fetus’ beating heart was visible on the ultrasound video for several seconds. Replicate measurements of cardiac contractions were averaged to determine the b.p.m. per exam.

Based on ultrasonography findings, profiles categorized as parturient were subclassified into term pregnancies (*n* = 6; profiles 1–6), in which all fetuses detected via ultrasonography were birthed, or partial loss (*n* = 2; profiles 7–8), in which two concepti were identified via ultrasound, but only one cub was birthed. Non-parturient females were subclassified as lost pregnancies (*n* = 2; profiles 9–10) when ultrasonography initially revealed a conceptus that was later undetectable with no evidence of birthing event or overt abortion, or pseudopregnant (*n* = 2; profiles 11–12), in which no conceptus was identified, but the female exhibited nesting behaviors similar to parturient females.

### Statistical analysis

All videos and images were reviewed and analyzed retrospectively by a single observer. Measurements of the uterine lumen were collected when the transducer was placed perpendicular to the uterine horn and opposing edges of the uterine lumen could be clearly visualized. In most species, day of pregnancy is reported relative to the day of ovulation; however, in species that undergo delayed implantation and have variable pregnancy lengths, like the red panda, day of pregnancy may be anchored to parturition date rather than breeding or ovulation date (red panda (Curry *et al.* 2017), giant panda (*Ailuropodamelanoleuca*) (Sutherland-Smith *et al.* 2004), and fishers (*Martespennanti*) (Frost *et al.* 2005)). Therefore, in parturient profiles, uterine lumen and fetal measurements were adjusted to the number of days until parturition (d 0). For the four non-parturient profiles, d 0 was calculated as the mean of all parturition events of this cohort (Curry *et al.* 2017). All values are reported as mean ± s.e.m. unless otherwise stated. Uterine lumen and fetal growth measurements were correlated to d 0 using simple linear regression analysis to determine growth rate.

## Results

### Breeding and parturition

Breeding was observed in five of eight (62.5%) parturient and two of four (50.0%) non-parturient profiles with a mean observed breeding date of February 11 ± 6.5 and a range of January 7 to March 22 (Table 1). In three profiles (25.0%), females were observed breeding multiple times during a breeding season. Parturition events occurred from June 6 and July 5, with a mean parturition date of June 16 ± 3.3 (Table 1). Pregnancy duration was 122.2 ± 7.3 d with a range of 107–149 d. Eight birthing events in four profiles resulted in twelve cubs, four of which were singletons (50.0%) and four were sets of twins (50.0%).

### Ultrasonography

#### Pregnancy diagnosis

The number of ultrasound examinations performed on each profile varied, with a mean of 9.1 ± 1.8 scans per profile and a range of 2–17 (Table 1). Because all examinations were conducted through voluntary participation of the animals, the duration and thoroughness of the scans depended on the individual’s cooperation and tolerance and thus, varied accordingly. Using ultrasonography, 16 concepti were detected in 10 of 12 profiles (83.3% pregnancy rate). Six (60.0%) were twin pregnancies and four (40%) singletons. No evidence of fetal tissue could be detected in the females considered pseudopregnant (*n* = 2; 16.7%), despite 15– 17 different scan dates.

#### Incidence of fetal loss

Loss of one or both concepti was documented in four of ten pregnancies (40%). Of the sixteen concepti identified, four (25.0%) were lost prior to parturition, two (20.0%) as partial losses, and two (20.0%) as full losses. No evidence of abortion or expulsion of fetus was observed grossly.

#### Timing of fetal loss

**Figure 2:**
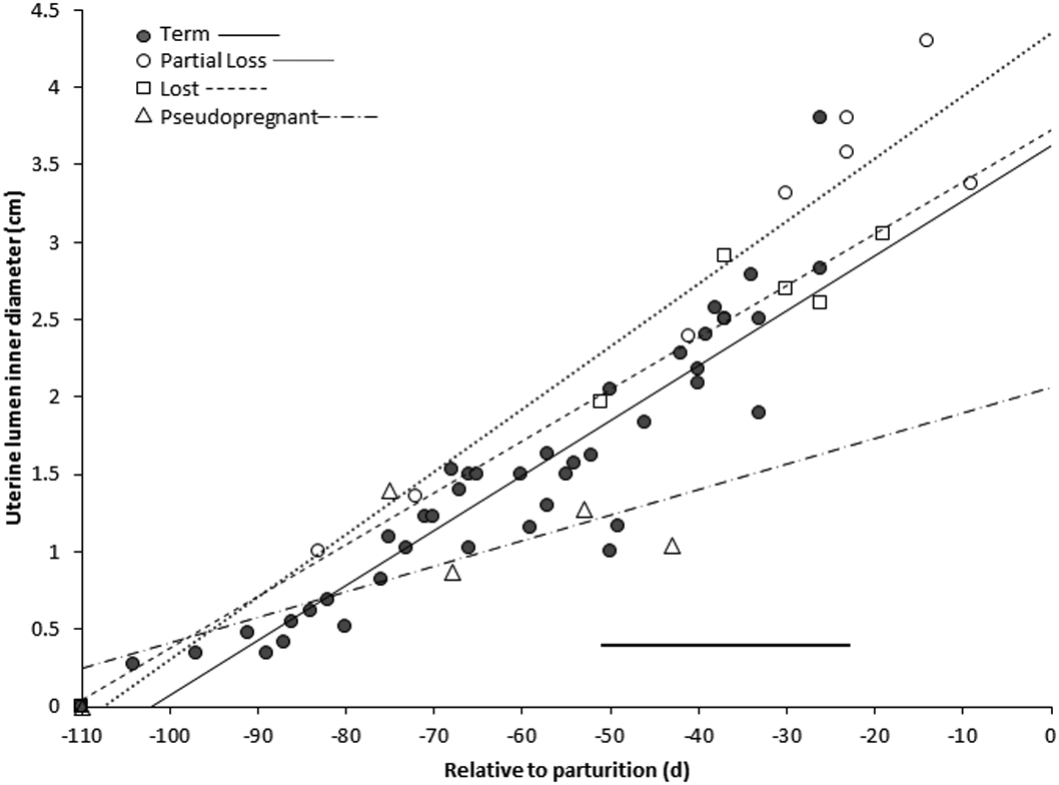
Growth of uterine lumen measured through trans-abdominal ultrasonography in term (*n* = 6), partial loss (*n* = 2), lost (*n* = 2), and pseudopregnant (*n* = 2) females in relation to date of parturition (d 0) (or mean date of parturition for non-parturient individuals). Trendlines for each group are included; however, there were no differences among term, partial loss, and lost pregnancies. Solid horizontal line indicates the range of fetal loss.

Two profiles exhibited loss of one conceptus of a twin pregnancy. In these partial loss pregnancies, two concepti were visible at d 37 or 27 pre-partum, but only one was discernible on d 23 pre-partum (Fig. 2), indicating loss occurred between d 37 and 23 in one pregnancy and between d 27 and 23 in the other. Each female gave birth to one cub. In two non-parturient profiles, a single conceptus was observed, but later was undetectable and no cubs were produced. In one lost pregnancy, the conceptus vanished between d 30 and 26 pre-partum, based on the average date of parturition, and in the other, was lost between d 51 and d 37.

#### Uterine growth

Uterine lumen diameters (*n* = 59; from eight parturient and four non-parturient females) were measured and graphed relative to parturition (Fig. 2). All profiles in which examinations were initiated early in the pregnancy had detectable fluid by d 83 pre-partum. The rate of uterine growth (0.04 cm/d) was similar among term, partial loss, and lost pregnancies (Fig. 2). In pseudopregnancies, the uterine lumen decreased in size 0.02 cm/d from d 53 to 43 pre-partum, based on the average date of parturition, and by d 39 could no longer be detected.

#### Fetal development

**Figure 3:**
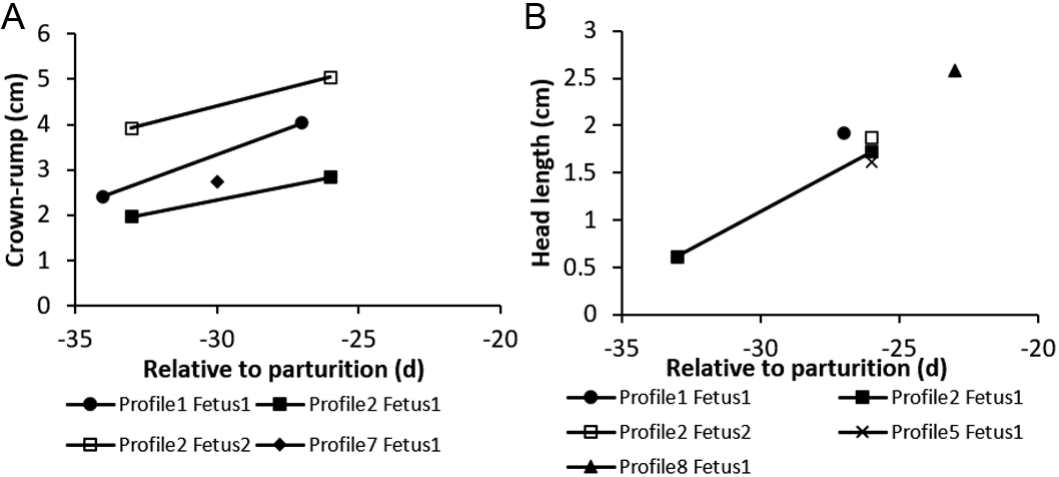
Crown-rump length and head length measurements relative to parturition (d 0). Panel A portrays fetal crown-rump length in four fetuses from three pregnancies (two singletons and one twin). Panel B shows fetal head length of five fetuses from four pregnancies (three singletons and one twin). One of the fetuses from profile 2 was stillborn.

The embryo proper could be identified as early as d 47.0 ± 3.5 pre-partum. At d 50 (d 62 post-breeding) and 40 days pre-partum (d 72 post-breeding), embryos measured 0.78 by 0.67 cm and 0.91 × 0.66 cm, respectively. Twins (*n* = 6) could be reliably detected at d 33.0 ± 3.0 pre-partum. Skeletal structures became visible around d 34 pre-partum coinciding with first signs of ossification. Discernable, but not fully ossified fetal structures (e.g. skull, body, and limb buds) were apparent with fetal movement observed on d 32.3 ± 1.2 (d 90.3 ± 14.4 post-breeding). By d 26.7 ± 0.3 (d 96.0 ± 13.1 post-breeding), the spine, ribs, limbs, and skull were easily discernible. By d 23, orbit and nasal cavities could be distinguished. Crown-rump length (*n* = 7) was obtained for four fetuses, and in three of these, additional measurements were acquired 7 d later, enabling calculation of the rate of fetal growth. On d 34/33, the mean crown-rump length was 2.76 ± 0.59 cm (range, 1.96–3.91 cm) and increased by 1.20 ± 0.05 cm 7 d later in three different fetuses (Fig. 3). Head length measurements (*n* = 6) were obtained from five fetuses in four pregnancies (Fig. 3). Head length was 0.61 cm at d 33, increased to 1.78 cm ± 0.07 at d 26/27, and was last measured at 2.58 cm at d 23, indicating a head length growth rate of 0.19 cm/day. A single cranial length measurement was obtained from three different fetuses between d 26 and d 23 pre-partum and averaged 1.39 ± 0.21 cm. Fetal heart rates of a single fetus in two partial loss pregnancies were 173 ± 4 b.p.m. (d 14 pre-partum) and 206 ± 2 b.p.m. (d 9 pre-partum).

## Discussion

Results of this retrospective analysis provide original insights into the rate and timing of pregnancy loss in red pandas. Of the ten pregnancies diagnosed via ultrasound, 20.0% were completely lost and 20.0% exhibited loss of one conceptus of a twin pregnancy, suggesting that spontaneous abortion may occur in ~40% of pregnancies. With the exception of ultrasonography, there is no proven method to diagnose and monitor pregnancy in this species; consequently, the incidence of fetal loss has been largely undetermined. Findings indicate that a substantial proportion of concepti is lost post-embryo reactivation and prior to parturition, which undoubtedly contributes to the relatively low offspring production rate of this species.

These findings revealed that pregnancies were lost between d 51 and 23 pre-partum. In two twin pregnancies, a single conceptus was lost between d 27–23 and between d 37–23 pre-partum, during the equivalent of the second trimester (d 40–20 pre-partum). Although the day of fetal loss relative to parturition could be determined in these partial loss pregnancies because females gave birth to another cub, accurate gestational age could not be assigned in the two lost profiles in which no parturition event occurred. Instead, fetal ages were calculated based upon the average parturition date for this cohort and uterine lumen sizes of the lost pregnancies were comparable to term pregnancies at the same timepoint. In lost profiles, the disappearance of the conceptus occurred between d 51 and 37 and, in the other, between d 30 and 26 pre-partum, which is similar to the timing in the partial loss pregnancies.

Because there was no evidence of expulsion of fetal tissue in any of the losses, it is likely that concepti were absorbed. There are reports of embryo resorption in many species, including canine (*Canis lupus familiaris*) (Sharma *et al.* 2018) and murine (*Musmusculus*) (Flores *et al.* 2014). In wolverine (*Gulogulo*) carcasses, examination of uteri revealed that resorption of fetuses was detected in 2 of 19 litters macroscopically; the authors hypothesized that non-detectable losses *in utero*, possibly during pre-implantation, are unknown but likely large (Banci & Harestad 1988). Dark-colored vaginal discharge and/or sharp reductions in progesterone secretion in spotted skunks (*Spilogaleputorius*) and ferrets (*Mustelaputorius*) in human care were often signs of embryo resorption (Mead *et al.* 1993), but in the current study, vaginal discharge was not observed, although it is possible it was undetected. In a giant panda (*Ailuropodamelanoleuca*), which has a post-implantation period of less than 50 d, twins were diagnosed via ultrasound, but one fetus presumedly died *in utero* between d 10 pre-partum and birthing (Sutherland-Smith *et al.* 2004). No fetal remnants were observed, suggesting that the fetus was resorbed. This timing of fetal loss suggests that resorption may occur even after ossification, as observed in the current study.

A limitation of the current study is that embryos in diapause are too small to be detected by ultrasonography and additional embryonic loss could be occurring during this initial phase of pregnancy. Therefore, females classified as pseudopregnant instead could be cases of early pregnancy loss if the embryos were lost during diapause. Around d 70 pre-partum, uterine lumen measurements in females classified as pseudopregnant were comparable to those of parturient females; however, it is unknown if uterine changes are a result of CL reactivation or the presence of an embryo. In species that experience delayed implantation, embryo reactivation is likely controlled by the reactivation of CL at the cessation of diapause; however, in pseudopregnant females, the CL secretes progesterone in concentrations similar to those observed in pregnant females (Hanssen 1947, Wei *et al.* 2005, Curry *et al.* 2017), so uterine changes induced by progesterone would be present regardless of the presence of an embryo. Methods to diagnose pregnancy during diapause would allow for discernment between true pregnancy and pseudopregnancy and would provide additional insight into the rate of early embryonic loss in this species. A proper control group would consist of females that were not housed with males but were induced to ovulate; however, because most reproductively viable females are recommended to breed, this was beyond the scope of this study.

Although there was some variation in uterine lumen size among term, partial loss, and lost pregnancies, there were no differences in uterine lumen size or growth rate during the interval studied, as depicted in Fig. 2. Consequently, it seems improbable that tracking uterine lumen growth would be useful in predicting whether an established pregnancy will result in loss. Conversely, in profiles assigned as pseudopregnancies, the echogenic uterine lumen increased in size from d 100 to 56 pre-partum (based on average parturition date) but then disappeared by ~d 40 and was no longer detectable. Therefore, a viable pregnancy diagnosis probably can be rejected if a uterine lumen is visible for a period of time and then later is indiscernible.

Results showed that general red panda developmental milestones were similar to domestic dogs (*Canis lupus familiaris*) and cats (*Feliscatus*), which have comparable durations of placental pregnancies of ~61 (Phemister *et al.* 1973, Pretzer 2008) and 65.6 d (Zambelli *et al.* 2002, Kustritz 2006), respectively. In red pandas, it was deduced that embryo reactivation likely occurs around d 60–65 pre-partum (Curry *et al.* 2017), and this study supports a red panda placental pregnancy of 60–65 d, as fetal developmental milestones are similar to other carnivores of similar size with comparable placental pregnancy lengths. The rate of fetal growth in red pandas appeared to be consistent among fetuses, but there was variability among measurements of individual fetuses obtained at similar timepoints. Two CRLs obtained on the same day were approximately 2 cm different between two fetuses in a twin pregnancy (profile 2); however, it should be noted that one cub was a stillborn, although it is unknown if the larger or smaller cub survived because comparative measurements were not taken at birth. Arguments could be made that a smaller cub was underdeveloped and died during birthing or that a larger cub may have led to dystocia, causing its fatality. At a comparable timepoint, the CRL from a term singleton pregnancy (profile 1 fetus 1) fell between the two measurements from the fetuses in profile 2. Head length between twins (profile 2) varied 0.15 cm and were both smaller than a term singleton pregnancy. A greater number of measurements from fetuses of singletons, twins, and triplets, both surviving and stillborn, are needed to establish standard reference ranges for this species.

Although not yet recognized as separate species, recent genomic evidence indicates there are actually two distinct red panda species: the Himalayan red panda (*Ailurusfulgens*) and the Chinese red panda (*Ailurusstyani*) (Hu *et al.* 2020) with *A.f. styani* being larger than *A.f. fulgens* (Groves 2011)*.* The morphology, skull size (Hu *et al.* 2020), body size (Groves 2011), and litter size (Northrop & Czekala 2011) differ, so it is plausible that there are differences in fetal sizes between *A.f. styani* and *A.f. fulgens* at comparable timepoints as well. Because the current analysis focused only on *A. f. styani*, fetal measurements may be slightly smaller for *A.f. fulgens*.

In conclusion, our results indicate that in this cohort of red pandas, 40% of pregnancies resulted in either partial loss or full loss and, of the total concepti identified via ultrasound, 25.0% were lost prior to parturition. Ultrasonography was useful for diagnosing pregnancy and the occurrence of fetal loss but did not indicate which pregnancies would lead to losses; however, pseudopregnancies could be identified by the presence and subsequent disappearance of fluid in the uterine lumen. Furthermore, the illumination of the rate and timing of pregnancy loss underscores the need to examine husbandry practices and environmental factors, especially in females that breed but fail to produce offspring. In most wildlife species, neither the rate nor timing of pregnancy loss is known, but animals housed in zoological settings provide unique opportunities to collect minimally invasive, longitudinal data to increase knowledge surrounding complex reproductive processes.

## Declaration of interest

The authors declare that there is no conflict of interest that could be perceived as prejudicing the impartiality of the research reported.

## Funding

This work did not receive any specific grant from any funding agency in the public, commercial, or not-for-profit sector.

## Author contribution statement

E C conceived the analysis, performed the ultrasound examinations, and provided guidance on data analysis. J L reviewed ultrasound video and images, recorded measurements, organized data, and created graphs. Authors contributed equally to writing the manuscript.
